# Supplementary material for: The Evolution of Covert Signaling
Source: Sci Rep. 2018 Mar 20;8:4905. doi: 10.1038/s41598-018-22926-1 (PMC5861109; doi:10.1038/s41598-018-22926-1)
Supplement: Supplementary file 1 — Supplementary Information [file 41598_2018_22926_MOESM1_ESM.pdf]

APPENDIX (ONLINE SUPPLEMENT)
THE EVOLUTION OF COVERT SIGNALING

PAUL E. SMALDINO[1], THOMAS J. FLAMSON[2], AND RICHARD MCELREATH[3]

Appendix A. Model derivation

A.1. **Payoff expressions.** As explained in the main text, we can define a general payoff expression for an individual with strategy p' and attitude matrix $\mathbf{a}'$ in a population with strategy $\{p, \mathbf{a}\}$. This expression is:

$$W(p', \mathbf{a}') = \Omega + q \Pr(\text{similar}|p', \mathbf{a}')+ \\ (1-q)\big(1 - \Pr(\text{Disliked}|p', \mathbf{a}')d - \Pr(\text{Disliked}|p', \mathbf{a}')\Pr(\text{Dislike}|p', \mathbf{a}')\delta\big) \quad (1)$$

where Ω is an expected baseline payoff due to other activities.

A.2. **Context 1 probabilities.** $\Pr(\text{similar}|p', \mathbf{a}')$ is the probability of ending up in a similar dyad, given the focal has signaling strategy $\{p', \mathbf{a}'\}$. By definition:

$$\Pr(\text{similar}|p', \mathbf{a}') = \Pr(\text{LL}|p', \mathbf{a}')\Pr(\text{similar}|\text{LL}, p', \mathbf{a}') + \Pr(\text{LN}|p', \mathbf{a}')\Pr(\text{similar}|\text{LN}, p', \mathbf{a}') \\ + \Pr(\text{NN}|p', \mathbf{a}')\Pr(\text{similar}|\text{NN}, p', \mathbf{a}')$$

The terms like $\Pr(\text{similar}|\text{NN}, p', \mathbf{a}')$ are defined by conditional probaility:

$$\Pr(\text{similar}|\text{NN}, p', \mathbf{a}') = \frac{\Pr(\text{similar}, \text{NN}|p', \mathbf{a}')}{\Pr(\text{NN}|p')}$$

Defining the two terms on the right requires defining probabilities for dyad formation.

The probability that an LL dyad forms is:

$$\Pr(\text{LL}|p', \mathbf{a}') = \frac{p(\text{LL}|p', \mathbf{a}')w_L^2}{Z}$$

where the denominator Z normalizes the probability and $p(\text{LL}|p', \mathbf{a}')$ is the raw proportion of dyads that are LL, post signaling. Under the baseline receiver strategy, $\underline{\mathbf{a}}'$, it is defined as:

$$p(\text{LL}|p', \underline{\mathbf{a}}') = s\big(p'pr^2 + (p'(1-p) + (1-p')p)rR + (1-p')(1-p)R^2\big)$$

While we later develop this expression in general for all receiver strategies, it's worth consider the specific expression above, for sake of comprehension. To understand this expression, consider that LL dyads must comprise similar individuals, and both signals have to be received for the attitudes to form. Individuals are similar s of the time. There are three ways this can happen: (1) both individuals signal covertly $p'p$ of the time, (2) one individual signals covertly and the other overtly $p'(1-p) + (1-p')p$ of the time, or (3) both individuals signal overtly $(1-p')(1-p)$ of the time. In all three cases, both signals must be received. Probabilities for each of the other dyad types—LN, NN, ND, and

[1]Cognitive and Information Sciences, University of California, Merced, Merced, CA, USA
[2]Independent Scholar, Sacramento, CA, USA
[3]Department of Human Behavior, Ecology and Culture, Max Planck Institute for Evolutionary Anthropology, Leipzig, Germany
E-mail address: paul.smaldino@gmail.com.

DD—are defined similarly. Further down, we define all of these probabilities in general, using a more algorithmic approach.

The denominator Z normalizes the probabilities of each dyad forming. It is merely the sum of all of the numerators in the probabilities of different types of dyads. These other probabilities are defined similarly:

$$\Pr(\text{LN}|p', \mathbf{a}') = p(\text{LN}|p', \mathbf{a}')w_L/Z$$
$$\Pr(\text{LD}|p', \mathbf{a}') = p(\text{LD}|p', \mathbf{a}')w_L w_D/Z$$
$$\Pr(\text{NN}|p', \mathbf{a}') = p(\text{NN}|p', \mathbf{a}')/Z$$
$$\Pr(\text{ND}|p', \mathbf{a}') = p(\text{ND}|p', \mathbf{a}')w_D/Z$$
$$\Pr(\text{DD}|p', \mathbf{a}') = p(\text{DD}|p', \mathbf{a}')w_D^2/Z$$

A.3. **Building generalized probabilities.** Now we construct the general probabilities that comprise the payoff expression by using a table of interactions.

[1]Focal	[2]Other	[3]Sim	[4]Signals	[5]Probability of dyad	[6]FL	[7]FN	[8]FD	[9]OL	[10]ON	[11]OD
C	C	1	11	$p'psr^2$	a'_{SL}	a'_{SN}	a'_{SD}	a_{SL}	a_{SN}	a_{SD}
C	C	1	10	$p'psr(1-r)$	a'_{NL}	a'_{NN}	a'_{ND}	a_{SL}	a_{SN}	a_{SD}
C	C	1	01	$p'ps(1-r)r$	a'_{SL}	a'_{SN}	a'_{SD}	a_{NL}	a_{NN}	a_{ND}
C	C	1	00	$p'ps(1-r)^2$	a'_{NL}	a'_{NN}	a'_{ND}	a_{NL}	a_{NN}	a_{ND}
C	C	0	11	$p'p(1-s)r^2$	a'_{NL}	a'_{NN}	a'_{ND}	a_{NL}	a_{NN}	a_{ND}
C	C	0	10	$p'p(1-s)r(1-r)$	a'_{NL}	a'_{NN}	a'_{ND}	a_{NL}	a_{NN}	a_{ND}
C	C	0	01	$p'p(1-s)(1-r)r$	a'_{NL}	a'_{NN}	a'_{ND}	a_{NL}	a_{NN}	a_{ND}
C	C	0	00	$p'p(1-s)(1-r)^2$	a'_{NL}	a'_{NN}	a'_{ND}	a_{NL}	a_{NN}	a_{ND}
C	O	1	11	$p'(1-p)srR$	a'_{SL}	a'_{SN}	a'_{SD}	a_{SL}	a_{SN}	a_{SD}
C	O	1	10	$p'(1-p)sr(1-R)$	a'_{NL}	a'_{NN}	a'_{ND}	a_{SL}	a_{SN}	a_{SD}
C	O	1	01	$p'(1-p)s(1-r)R$	a'_{SL}	a'_{SN}	a'_{SD}	a_{NL}	a_{NN}	a_{ND}
C	O	1	00	$p'(1-p)s(1-r)(1-R)$	a'_{NL}	a'_{NN}	a'_{ND}	a_{NL}	a_{NN}	a_{ND}
C	O	0	11	$p'(1-p)(1-s)rR$	a'_{DL}	a'_{DN}	a'_{DD}	a_{NL}	a_{NN}	a_{ND}
C	O	0	10	$p'(1-p)(1-s)r(1-R)$	a'_{NL}	a'_{NN}	a'_{ND}	a_{NL}	a_{NN}	a_{ND}
C	O	0	01	$p'(1-p)(1-s)(1-r)R$	a'_{DL}	a'_{DN}	a'_{DD}	a_{NL}	a_{NN}	a_{ND}
C	O	0	00	$p'(1-p)(1-s)(1-r)(1-R)$	a'_{NL}	a'_{NN}	a'_{ND}	a_{NL}	a_{NN}	a_{ND}
O	C	1	11	$(1-p')psRr$	a'_{SL}	a'_{SN}	a'_{SD}	a_{SL}	a_{SN}	a_{SD}
O	C	1	10	$(1-p')psR(1-r)$	a'_{NL}	a'_{NN}	a'_{ND}	a_{SL}	a_{SN}	a_{SD}
O	C	1	01	$(1-p')ps(1-R)r$	a'_{SL}	a'_{SN}	a'_{SD}	a_{NL}	a_{NN}	a_{ND}
O	C	1	00	$(1-p')ps(1-R)(1-r)$	a'_{NL}	a'_{NN}	a'_{ND}	a_{NL}	a_{NN}	a_{ND}
O	C	0	11	$(1-p')p(1-s)Rr$	a'_{NL}	a'_{NN}	a'_{ND}	a_{DL}	a_{DN}	a_{DD}
O	C	0	10	$(1-p')p(1-s)R(1-r)$	a'_{NL}	a'_{NN}	a'_{ND}	a_{DL}	a_{DN}	a_{DD}
O	C	0	01	$(1-p')p(1-s)(1-R)r$	a'_{NL}	a'_{NN}	a'_{ND}	a_{NL}	a_{NN}	a_{ND}
O	C	0	00	$(1-p')p(1-s)(1-R)(1-r)$	a'_{NL}	a'_{NN}	a'_{ND}	a_{NL}	a_{NN}	a_{ND}
O	O	1	11	$(1-p')(1-p)sR^2$	a'_{SL}	a'_{SN}	a'_{SD}	a_{SL}	a_{SN}	a_{SD}
O	O	1	10	$(1-p')(1-p)sR(1-R)$	a'_{NL}	a'_{NN}	a'_{ND}	a_{SL}	a_{SN}	a_{SD}
O	O	1	01	$(1-p')(1-p)s(1-R)R$	a'_{SL}	a'_{SN}	a'_{SD}	a_{NL}	a_{NN}	a_{ND}
O	O	1	00	$(1-p')(1-p)s(1-R)^2$	a'_{NL}	a'_{NN}	a'_{ND}	a_{NL}	a_{NN}	a_{ND}
O	O	0	11	$(1-p')(1-p)(1-s)R^2$	a'_{DL}	a'_{DN}	a'_{DD}	a_{DL}	a_{DN}	a_{DD}
O	O	0	10	$(1-p')(1-p)(1-s)R(1-R)$	a'_{NL}	a'_{NN}	a'_{ND}	a_{DL}	a_{DN}	a_{DD}
O	O	0	01	$(1-p')(1-p)(1-s)(1-R)R$	a'_{DL}	a'_{DN}	a'_{DD}	a_{NL}	a_{NN}	a_{ND}
O	O	0	00	$(1-p')(1-p)(1-s)(1-R)^2$	a'_{NL}	a'_{NN}	a'_{ND}	a_{NL}	a_{NN}	a_{ND}

The columns of this table specify:

(1) The focal individual's signaling strategy
(2) The other individual's signaling strategy
(3) Whether or not (0/1) the individual's are similar
(4) Whether or not (0/1) focal/other signals are received by the other individual in the pair. 11 indicates that both signals are received. 10 indicates that focal's signal was received, while other's signal was not.
(5) The probability of this pairing and pair of signal reception events
(6) The probability that the focal individual forms attitude L. When a signal is received from a similar individual, this is a'_{SL}. When no signal is received or a covert signal is received from a dissimilar individual, this is a'_{NL}. When an overt signal is received from a dissimilar individual, this is a'_{DL}.
(7) The probability that the focal individual forms attitude N
(8) The probability that the focal individual forms attitude D

(9) The probability that the other individual forms attitude L
(10) The probability that the other individual forms attitude N
(11) The probability that the other individual forms attitude D

Parameters marked by a prime, such as p' and a'_{SL}, indicate aspects of the focal individual's strategy, to be contrasted with population values. Again, we refer to the vector of attitude parameters with $\mathbf{a}$.

Call this table $\mathbf{M}$. To compute probabilities, we multiply specific terms in each row and then sum these products down the rows. For example, the probability that the focal individual and a random individual mutually like one another, $p(\text{LL}|p', \mathbf{a}')$, is defined by:

$$p(\text{LL}|p', \mathbf{a}') = \textstyle\sum_{i=1}^{32} \mathbf{M}_{i,5}\mathbf{M}_{i,6}\mathbf{M}_{i,9} \tag{2}$$

The other probabilities are defined similarity:

$$p(\text{LN}|p', \mathbf{a}') = \textstyle\sum_{i=1}^{32} \mathbf{M}_{i,5}(\mathbf{M}_{i,6}\mathbf{M}_{i,10} + \mathbf{M}_{i,7}\mathbf{M}_{i,9}) \tag{3}$$

$$p(\text{LD}|p', \mathbf{a}') = \textstyle\sum_{i=1}^{32} \mathbf{M}_{i,5}(\mathbf{M}_{i,6}\mathbf{M}_{i,11} + \mathbf{M}_{i,8}\mathbf{M}_{i,9}) \tag{4}$$

$$p(\text{NN}|p', \mathbf{a}') = \textstyle\sum_{i=1}^{32} \mathbf{M}_{i,5}\mathbf{M}_{i,7}\mathbf{M}_{i,10} \tag{5}$$

$$p(\text{ND}|p', \mathbf{a}') = \textstyle\sum_{i=1}^{32} \mathbf{M}_{i,5}(\mathbf{M}_{i,7}\mathbf{M}_{i,11} + \mathbf{M}_{i,8}\mathbf{M}_{i,10}) \tag{6}$$

$$p(\text{DD}|p', \mathbf{a}') = \textstyle\sum_{i=1}^{32} \mathbf{M}_{i,5}\mathbf{M}_{i,8}\mathbf{M}_{i,11} \tag{7}$$

To derive probabilities of similarity and attitudes, all that is required is to multiply each of the products above with the corresponding value in column 3. This defines:

$$\Pr(\text{sim}, \text{LL}|p', \mathbf{a}') = \textstyle\sum_{i=1}^{32} \mathbf{M}_{i,3}\mathbf{M}_{i,5}\mathbf{M}_{i,6}\mathbf{M}_{i,9} \tag{8}$$

$$\Pr(\text{sim}, \text{LN}|p', \mathbf{a}') = \textstyle\sum_{i=1}^{32} \mathbf{M}_{i,3}\mathbf{M}_{i,5}(\mathbf{M}_{i,6}\mathbf{M}_{i,10} + \mathbf{M}_{i,7}\mathbf{M}_{i,9}) \tag{9}$$

$$\Pr(\text{sim}, \text{LD}|p', \mathbf{a}') = \textstyle\sum_{i=1}^{32} \mathbf{M}_{i,3}\mathbf{M}_{i,5}(\mathbf{M}_{i,6}\mathbf{M}_{i,11} + \mathbf{M}_{i,8}\mathbf{M}_{i,9}) \tag{10}$$

$$\Pr(\text{sim}, \text{NN}|p', \mathbf{a}') = \textstyle\sum_{i=1}^{32} \mathbf{M}_{i,3}\mathbf{M}_{i,5}\mathbf{M}_{i,7}\mathbf{M}_{i,10} \tag{11}$$

$$\Pr(\text{sim}, \text{ND}|p', \mathbf{a}') = \textstyle\sum_{i=1}^{32} \mathbf{M}_{i,3}\mathbf{M}_{i,5}(\mathbf{M}_{i,7}\mathbf{M}_{i,11} + \mathbf{M}_{i,8}\mathbf{M}_{i,10}) \tag{12}$$

$$\Pr(\text{sim}, \text{DD}|p', \mathbf{a}') = \textstyle\sum_{i=1}^{32} \mathbf{M}_{i,3}\mathbf{M}_{i,5}\mathbf{M}_{i,8}\mathbf{M}_{i,11} \tag{13}$$

All that remains are probabilities that any random individual Likes or Dislikes the focal:

$$\Pr_F(\text{L}|p', \mathbf{a}') = \textstyle\sum_{i=1}^{32} \mathbf{M}_{i,5}\mathbf{M}_{i,9} \tag{14}$$

$$\Pr_F(\text{D}|p', \mathbf{a}') = \textstyle\sum_{i=1}^{32} \mathbf{M}_{i,5}\mathbf{M}_{i,11} \tag{15}$$

as well as the probabilities that the focal likes or dislikes the other individual:

$$\Pr_O(\text{L}|p', \mathbf{a}') = \textstyle\sum_{i=1}^{32} \mathbf{M}_{i,5}\mathbf{M}_{i,6} \tag{16}$$

$$\Pr_O(\text{D}|p', \mathbf{a}') = \textstyle\sum_{i=1}^{32} \mathbf{M}_{i,5}\mathbf{M}_{i,8} \tag{17}$$
